# What Is Cholera?

**Published:** 1893-06

**Authors:** William Watson Hall


### WHAT IS CHOLERA?

The theory of cholera is, in its nature, common diarrhœa intensified, just as yellow fever is an intensification of common bilious fever—a concentrated form of it. But what causes this loose condition of the bowels, which is not, indeed, a premonitory symptom of cholera, but which is cholera itself? That which precedes the loose bowels of diarrhœa and cholera is liver inaction; the liver is torpid, that is, it does not abstract the bile from the blood, or if it does, this bile, instead of being discharged drop by drop from the gall bladder into the top or beginning of the intestines, where the food passes out of the stomach into the bowels proper, is retained and more or less reabsorbed and thrown into the general circulation, rendering it every hour thicker and thicker, and more and more impure and black, until at length it almost ceases to flow through the veins, just as water will very easily pass along a hose pipe or hollow tube, while mush or stirabout would do so with great difficulty; and not passing out of the veins, but still coming in, the veins are at length so much distended that the thinner portions ooze through the blood vessels. That which oozes through the blood vessels on the inner side of the stomach and bowels is but little more than water, and constitutes the rice water discharges so much spoken of in this connection, that which oozes through the blood vessels on the surface constitutes the sweat which bedews the whole body shortly before death, and it is this clogging up of the thick black blood in the small veins which gives the dark blue appearance of the skin in the collapsed stage. What is the reason that the liver is torpid, does not work,—does not withdraw the bile from the blood?

It is because the blood has become impure, and being thus when it enters the liver it fails to produce the natural stimulus and thus does not wake it up to its healthful action, just as the habitual drinker of the best brandy fails to be put "in usual trim" by a "villainous article."

But how does the blood become impure? It becomes impure by there being absorbed into the circulation what some call malaria, and others call miasm.

But by whatever name it may be called, this death-dealing substance is a gas arising from the combination of three substances, heat, moisture and vegetation. Without these three things in combination there can be no "cholera atmosphere," there can be no epidemic cholera in these ages of the world. Vegetable matter decomposes at a heat of between seventy and eighty degrees, and that amount of heat in combination with moisture and some vegetable substance must always precede epidemic cholera. The decomposition in burial grounds, in potters'

fields, or of animal matter in any stage or form, does not excite or cause cholera; if anything, it prevents it. I have no disposition to argue upon these points. I merely give them as my views, which I think, time and just observation will steadily corroborate.

The reader may think that he could state some strong facts in contradiction of those given, but I think it quite likely that on investigation these facts of his will be corroborants. For example; how is it that cholera has raged in latitudes where snow is on the ground five or ten feet deep? The people in such countries are generally poor, myriads of them live in snow houses, which are large spaces dug in the snow, with no outlet but one for the smoke, and in this house they live with their domestic animals and all the family offal for months together, so that in the spring of the year there is a crust of many inches of made flooring, while the interior heat from their own bodies, and from the fire for cooking purposes is often eighty or ninety degrees.

### THE THEORY OF CURE.

I have said that a torpid liver is an immediate cause of cholera, that it does not work actively enough to separate the bile, the impure particles from the blood.

Whatever then wakes up the liver, removes the torpidity or in plain language, whatever stimulates the liver to greater activity, that is curative of cholera.

Calomel is a medicine which acts upon, which stimulates the liver to action with a promptness and certainty infinitely beyond all the other remedies yet known to men, and the use of any other medicine as a substantive in any plain case of cholera, is in my opinion a trifling with human life; not that other remedies are not successful, but that this is more certain to act upon the liver than all others; and what sensible man wants to try a lesser certainty in so imminent a danger. My whole view as to cholera and calomel is simply this, that while cholera is arrested and cured by a variety of other agents, calomel will cure in all these and thousands of others where other remedies have no more effect than a thimblefull of ashes; that calomel will cure any case of cholera which any other remedy cures and that it will cure millions of other cases which no other remedy can reach; that when calomel fails to cure, all other things will inevitably fail.

### PREVENTIVE OF CHOLERA.

There are none, there never can be, except so far as it may be done by quietude of body and mind, by personal cleanliness, by regular and temperate habits of life and the use of plain accustomed nourishing food. Anything taken medicinally as a preventive of cholera will inevitably, and under all circumstances, increase the liability to an attack.

### WHY ?

Nothing can prevent cholera in a cholera atmosphere, beyond the natural agents of nutrition, except in proportion to its stimulating properties. The liver takes its share of the general stimulus and works with more vigor. When the system is under the effect of the stimulus, it is safer, but it is a first truth that the stimulant sooner or later expends its force, as a drink of brandy, for example. That moment the system begins to fail and falls as far below its natural condition as it was just before above it, and while in that condition is just as much more susceptible of cholera as it was less liable under the action of the stimulant, until by degrees it rises up to its natural equilibrium, its natural condition. You can, it is true, repeat the stimulus, but it must be done with the utmost regularity, and just at the time the effect of the previous one begins to subside. This, it will at once be seen requires a nicety of observation, and correctness of judgment which not one in a multitude can bestow, saying nothing of another nicety of judgment, that of gradually increasing the amount of the stimulant, so that the effect shall be kept up to the regular notch ; for a given amount of one stimulant will inevitably fail, after a few repetitions, to produce the same amount of stimulation and the moment that amount fails to be raised, that moment the person is more susceptible of cholera than if he had taken nothing at all. He who takes any medicinal agent, internal or external, for the prevention of cholera, commits an act of the most consummate folly ; and I should consider myself an ignoramus or a knave were I to concoct a professed anti-cholera mixture.

### THE SUMMING UP.

When cholera is present in any community, each person should consider himself as attacked with cholera,

First.—If the bowels act less frequently than usual.

Second.—If the bowels act oftener than twice in twenty-four hours.

Third.—If the discharge of the bowels is of a dirty white in color, and watery in its substance.

If he have any indefinable sensation about the belly, which not only unpleasantly reminds him that he has such an article, but also inclines him to sit down, and makes sitting down a much more pleasant operation than usual. Some persons may think that this fourth item is putting "too fine a point" on the matter, and that it is being over careful; but I know that these very feelings do, in a vast majority of fatal cases of cholera, precede the actual "looseness" so universally and so wrongfully regarded as the premonitory symptom of cholera; "looseness" is not a premonitory sympton of cholera.

Whenever cholera is prevalent in any community, it is as much actual cholera, under such circumstances, as the first little flame on the roof of a house constitutes a "house on fire."

When cholera is present as an epidemic, a person may have one regular action every twenty-four hours; if this action is followed by unpleasant sensations not relieved until the body is in a bent condition, these are the premonitions of Asiatic cholera; and it is wonderful that they have never, as far as I know, been published in a book or newspaper for popular information. At such a stage no physician is needed, no physic is required, only quietude on the back, ice to be eaten, if there is any thirst, and no food but toasted bread and tea of some kind, green, black, sage, sassafras, or any other of the common herbs. Keep up attention to these things until you can walk without any uncomfortableness whatever, and even feel as if it were doing you good and until you are not sensible of anything unpleasant about the stomach. If you get tired of tea and toast, or if it is not agreeable to you, use in their place boiled rice, or sago, or tapioca, or arrow root, or corn starch, or mush made of rice flour. With all of these articles a little boiled milk may be used, or they may be eaten with a little butter or syrup of some kind for a change.

The most certain indication of recovery from an attack of Asiatic cholera is the return of free urination; for during the attack it ceases altogether, a most important fact, but not known, perhaps, to one person in ten thousand, and is worth more than all other symptoms together.

WILLIAM WATSON HALL, M. D.

To be Concluded.

